# Rate and Temporal Coding Convey Multisensory Information in Primary Sensory Cortices

**DOI:** 10.1523/ENEURO.0037-17.2017

**Published:** 2017-03-20

**Authors:** Malte Bieler, Kay Sieben, Nicole Cichon, Sandra Schildt, Brigitte Röder, Ileana L. Hanganu-Opatz

**Affiliations:** 1 Developmental Neurophysiology, Institute of Neuroanatomy, University Medical Center Hamburg-Eppendorf, Hamburg 20251, Germany; 2 Biological Psychology and Neuropsychology, University Hamburg, Hamburg 20146, Germany

**Keywords:** multisensory, neuronal firing, oscillation, primary visual cortex, barrel field, rate coding, temporal coding

## Abstract

Optimal behavior and survival result from integration of information across sensory systems. Modulation of network activity at the level of primary sensory cortices has been identified as a mechanism of cross-modal integration, yet its cellular substrate is still poorly understood. Here, we uncover the mechanisms by which individual neurons in primary somatosensory (S1) and visual (V1) cortices encode visual-tactile stimuli. For this, simultaneous extracellular recordings were performed from all layers of the S1 barrel field and V1 in Brown Norway rats *in vivo* and units were clustered and assigned to pyramidal neurons (PYRs) and interneurons (INs). We show that visual-tactile stimulation modulates the firing rate of a relatively low fraction of neurons throughout all cortical layers. Generally, it augments the firing of INs and decreases the activity of PYRs. Moreover, bimodal stimulation shapes the timing of neuronal firing by strengthening the phase-coupling between neuronal discharge and theta–beta band network oscillations as well as by modulating spiking onset. Sparse direct axonal projections between neurons in S1 and V1 seem to time the spike trains between the two cortical areas and, thus, may act as a substrate of cross-modal modulation. These results indicate that few cortical neurons mediate multisensory effects in primary sensory areas by directly encoding cross-modal information by their rate and timing of firing.

## Significance Statement

To optimally interact with the environment, diverse sensory inputs need to be bound together within a unified percept. This process takes place in putatively unisensory neocortical areas. However, the strategies of multisensory coding at the neuronal level remain largely unknown. Here, we show that neurons in primary sensory cortices use a dual code for conveying simultaneous visual and tactile stimuli. First, visual-tactile stimulation affects the firing rates of a small population of pyramidal neurons (PYRs) and interneurons (INs). Second, it enhances the firing precision of individual neurons by augmenting the phase locking to network oscillations and the coupling strength between spike trains. These data identify rate and temporal coding as neuronal mechanisms of multisensory processing in primary sensory cortices.

## Introduction

Survival and appropriate behavior require constant integration of a multitude of sensory inputs from the environment. As a result, stimulus detection and reaction times improve (Gielen et al., 1983; Driver and Spence, 1998; Gleiss and Kayser, 2012). This combinatorial processing of information takes place not only in higher association areas, but also, as recently demonstrated, in putatively unisensory areas, such as primary sensory cortices (Ghazanfar and Schroeder, 2006; Macaluso, 2006; Lakatos et al., 2007; Driver and Noesselt, 2008; Kayser et al., 2008; Sieben et al., 2013). Modulation of network oscillations in their power and phase has been found to represent a powerful mechanism of cross-modal integration (Lakatos et al., 2007; Sieben et al., 2013). It seems to use as anatomic substrate not only thalamo-cortical projections (Zikopoulos and Barbas 2007, Lakatos et al., 2009) but also direct axonal projections between primary sensory cortices that have been documented across multiple species and areas (Falchier et al., 2002; Budinger et al., 2006; Hall and Lomber, 2008; Sieben et al., 2013; Stehberg et al., 2014; Zingg et al., 2014; Sieben et al., 2015).

Despite augmenting evidence for the role of primary sensory cortices in multisensory processing, it is still largely unknown how individual neurons in these areas encode the information content of multiple senses. Most of the knowledge comes from the auditory system where visual and/or tactile stimuli modulate the neuronal firing, in most cases by suppressing its discharge (Bizley et al., 2007; Kayser et al., 2008; Meredith and Allman, 2015). These effects critically depend on the precision of spike timing. Dissection of underlying microcircuits revealed that interareal synaptic inhibition augments the salience of relevant stimuli by degrading the potentially distracting sensory processing (Iurilli et al., 2012; Olcese et al., 2013l; Ibrahim et al., 2016). To which extent these cellular rules of multisensory integration are common for all primary sensory cortices is still largely unknown.

Generally, neurons carry information about modality-specific sensory stimuli by using either a firing rate code (i.e., neurons modulate their action potential frequency when the “preferred” stimulus is presented) or a temporal code (i.e., sharpening of the coincidence of spiking) (Masuda and Aihara, 2007; Ainsworth et al., 2012; Panzeri et al., 2015). While the two coding mechanisms may occur separately (Roelfsema et al., 2004; Womelsdorf and Fries, 2006), in most experiments, changes both in rate and spike timing/correlation have been described (Biederlack et al., 2006; Zuo et al., 2015). Similar dual coding mechanisms by individual neurons might underlie multisensory communication at the level of primary sensory cortices by which the salience of a certain stimulus and, thereby, its behavioral impact are augmented.

To test this hypothesis, we focused on the cellular mechanisms underlying visual-somatosensory interactions at the level of primary sensory cortices. We provide electrophysiological and anatomic evidence that simultaneous visual and tactile stimuli modulate the firing rate and onset of a small population of cortical neurons. Moreover, cross-modal stimulation strengthens the phase-coupling of neuronal firing to network oscillations and the synchrony between spike trains. Anatomic evidence suggests that direct corticocortical axonal projections underlie these effects.

## Materials and Methods

### Subjects

The experiments were conducted in compliance with the German laws and the guidelines of the European Community for the use of animals in research and were approved by the local ethical committee (21/10 and 95/15). Brown Norway rats of both sexes (*n* = 24, weight 32-45 g at time of surgery) were obtained from Charles River, housed individually in the animal facility of the University Medical Center with a 12/12 h light/dark cycle and fed *ad libitum*. All experiments were conducted during the light phase under sleep-like conditions mimicked by urethane anesthesia (Bitzenhofer et al., 2015). By these means, the interference with spontaneous whisking and the impact of alert state, which modulate cross-modal integration, were avoided. However, we cannot exclude that this experimental control came at the cost of more single-unit responsiveness to bimodal stimulation (Populin, 2005).

### Surgery

The surgery was performed under ketamine/xylazine anesthesia (72/9.6 mg/kg body weight, i.p.; Ketavet; Rompun), and respiratory rate as well as pain reflexes were monitored. A circumscribed area of the scalp was removed, the neck muscles were detached from the skull, and two metal anchor bars were fixed on the nasal and occipital region with dental cement. Small parts of the skull were removed by drilling holes (0.5 mm in diameter) to expose the dura without causing leakage of cerebrospinal fluid or blood over primary somatosensory (S1) and visual (V1) cortices. The rat’s eyes were covered with ointment (Bepanthen), and the ear canals were filled with silicon adhesive (Kwik-Sil, World Precision Instruments) to block auditory input.

### Recording protocols

Extracellular recordings of the local field potential (LFP) and multiunit activity (MUA) were performed from head-fixed rats under light urethane anesthesia (0.5 g/kg body weight, i.p.; Sigma-Aldrich) using one-shank 16-channel electrodes (0.5-3 MΩ Silicon Michigan probes, Neuro-nexus Technologies; 100-μm intersite spacing) that were inserted into S1 barrel field (2.4-2.6 mm posterior and 5.5-5.8 mm lateral to bregma) and V1 (6.9-7.1 mm posterior and 3.4-3.7 mm lateral to bregma). Electrodes spanned supragranular, granular, and infragranular layers and were labeled with DiI (1,1’-dioctadecyl-3,3,3’,3’-tetramethyl indocarbocyanine; Invitrogen) for postmortem reconstruction of their tracks in histologic sections. A silver wire was inserted into the cerebellum and served as ground and reference electrode. The body temperature of the animal was kept constant at 37°C during recording. The electrical activity was recorded at a sampling rate of 32 kHz using a multichannel extracellular amplifier (no gain, Digital Lynx 10S, Neuralynx) and the acquisition software Cheetah.

### Sensory stimulation

Unimodal (either light flash or whisker deflection) or bimodal (simultaneous light flash and whisker deflection) stimuli were applied using a custom-made stimulation device as previously described (Sieben et al., 2013; Sieben et al., 2015). Briefly, whiskers were stimulated by deflection through compressed air-controlled roundline cylinders (RT/57110/M/10, Norgren) gated via solenoid valves (VCA, SMCPneumatik). The device produced almost silent, nonelectrical stimulation with precise timing (0.013 ± 0.81 ms) that was constant over all trials/conditions. For full eye field visual stimulation, 50-ms-long LED light flashes (300 lux) were used. For bimodal stimulation, whisker deflection and light flashes were applied in the same hemifield. The stimuli were randomly presented in three different stimulation conditions (unimodal tactile, unimodal visual, bimodal visual-tactile). Each type of stimulus was presented 100 times contralateral to the recording electrodes with an interstimulus interval of 6.5 ± 0.5 s. To achieve a physically simultaneous stimulation of whiskers (valve-controlled whisker stimulation) and eyes (instantaneous light flash), the time delay of whisker stimulation was calculated to match visual stimulation onset. The nonstimulated eye was covered with an aluminum foil patch.

### Retrograde tracing and immunohistochemistry

Retrograde tracer injections were performed as previously described (Sieben et al., 2015). In brief, ketamine/xylazine anesthetized rats were immobilized into a preformed mold fixed into the stereotaxic apparatus and received unilateral injections of the retrograde tracer Fluorogold (FG; Fluorochrome) in S1 barrel field (2.4-2.6 mm posterior and 5.5-5.8 mm lateral to bregma) or V1 (6.9-7.1 mm posterior and 3.4-3.7 mm lateral to bregma). A total volume of 100 nl FG (5% in dH_2_O) was injected (30 nl/min) via a 26-G needle attached to a pump controller (Micro4, World Precision Instruments) at a cortical depth of 300 µm. The syringe was left in place for 3 min to ensure an optimal diffusion of the tracer. The surgical opening was sealed with fibrin glue (Surgibond, SMI sutures) and postsurgery analgesic therapy was given (Meloxicam; 0.1-0.2 mg/kg). After a survival time of 4-8 d, the animals were deeply anesthetized with ketamine/xylazine and perfused transcardially with 4% paraformaldehyde (PFA). Brains were removed and postfixed in 4% PFA for 24 h. Coronal slices were sectioned at 50 µm and treated with PBS containing 0.2% Triton X-100 (Sigma-Aldrich), 10% normal bovine serum (Jackson ImmunoResearch), and 10% donkey serum (Millipore). The sections were incubated 2-4 d with mouse monoclonal Alexa Fluor 488-conjugated antibody against NeuN (1:100, MAB377X, Millipore) and rabbit polyclonal primary antibody against GABA (1:1000, #A2052, Sigma-Aldrich) followed by a 2-h incubation with Alexa Fluor 568 donkey anti-rabbit IgG secondary antibody (1:1000, A10042, Invitrogen).

Fluorescent images were obtained with a Axioskop 2 Mot microscope (Zeiss) equiped with a fluorescence camera. For quantification of retrogradely backlabeled cells, five 50 µm thick sections spanning S1 and V1 were selected and regions of interests (ROIs; height: 150 µm, width: 300 µm) were defined using ImageJ software. FG- and GABA-positive neurons were counted within each ROI and normalized to the number of NeuN-positive cells detected within supragranular, granular, and infragranular layers.

Fluorescent Nissl staining was performed as previously described (Brockmann et al., 2011) using NeuroTrace 500/525 green fluorescent Nissl stain (Invitrogen). Coronal sections were incubated for 20 min with 1:100 diluted NeuroTrace (Thermo Fisher), washed, and coverslipped with Fluoromount-G (SouthBiotech). To precisely detect the position of DiI-stained electrode, the sections were examined using the green (460-488 nm) and red (535-555 nm) excitation filters of the fluorescence microscope (Imager M1, Zeiss). The photographs were adjusted for brightness and contrast using Adobe Photoshop (version CS6).

### Data analysis

Data were imported and analyzed offline using custom-written tools in Matlab software version R2013B (MathWorks). For antialiasing, the signal was bandpass filtered (0.1 Hz and 5 kHz) by the Neuralynx recording system. A third-order Butterworth filter was applied. LFP data were down-sampled by a factor of 10.

#### Spike sorting and cluster analysis

The position of recording sites over layers was confirmed by electrophysiological (i.e., reversal of the evoked potential between supragranular and granular layers) and histologic (i.e., granular cell body layer) landmarks. Recording sites within the transition between cortical layers were not considered for analysis. The raw signal was high-pass filtered (>400 Hz). The threshold for detecting MUA was set individually at 25-30 µV. The stored signals were sorted offline depending on waveform shapes using spike sorting software (Plexon). A group of similar waveforms was considered as being generated from a single neuron if it defined a discrete cluster in a 2D/3D space and exhibited a clear refractory period (>1 ms) in the interspike interval histogram. The quality of separation between identified clusters was assessed by four different statistical measurements: the classical parametric F statistic of multivariate ANOVA (MANOVA), the J3 and PseudoF (Psf) statistics, and the Davies-Bouldin validity index (DB) (Davies and Bouldin, 1979; Späth, 1980). The values of statistical testing ranged between 8.55623e-007 and 0.1 for MANOVA, 0.85 and 11.19 for J3, 554 and 12833 for PsF, and 0.19 and 3.45 for DB. A total number of 262 units were clustered in S1, whereas a total number of 246 units were identified in V1. Approximately one to three units per recording site could be detected in each cortical layer (supragranular, granular, infragranular). To classify the units into pyramidal neurons (PYRs) or interneurons (INs), four characteristic features of the extracted waveforms were used: (1) spike duration, (2) spike after-hyperpolarization duration, (3) spike end slope, and (4) spike trough-to-peak duration. The feature values of all units over all layers were used for principal component (PC) analysis. For S1 units, the first three PCs accounted for 99.5% of the variance (PC1 66.5%, PC2 22.3%, PC3 10.7%), while for units in V1, the first three PCs accounted for 99.0% of the variance (PC1 76.1%, PC2 15.0%, PC3 8.7%). A k-means cluster algorithm (k = 2) was applied to the first three PCs to classify the sorted units.

#### Firing rate

MUA and single-unit activity (SUA) were calculated before and after stimulus (MUA: ± 1 s, 10 ms bin size; SUA: ± 1 s, 3 ms bin size) and summed over trials. Units were considered as being responsive if the stimulus-induced firing response was significantly modified, e.g., it exceeded 1.96 times the SD [95% confidence interval (CI)] of the spontaneous firing rate averaged 1–0.9 s before stimulus. To categorize the units that display a significant change in firing, their rate of discharge was calculated as number of spikes during the first time interval (0-80 ms) after modality-specific unimodal stimulation (i.e., tactile stimulation for units in S1 or visual stimulation for units in V1) and compared with the spiking response to unimodal but modality-unspecific stimulation (i.e., tactile stimulation for units in V1 or visual stimulation for units in S1) as well as to bimodal stimulation (i.e., visual-tactile stimulation for units in S1 or V1). The latency of SUA was measured using the average first-spike latency across trials. The MUA peak of firing was obtained by narrowing the bin size to 1/sampling rate and detecting subsequently the bin with maximum firing rate.

#### Multisensory interactions

In line with previously established criteria (Kayser et al., 2008), single units were classified into five groups according to the responsiveness within the first 100 ms to unimodal and bimodal stimulation (see Figure 2*E*, i and ii): (1) unimodal, (2) cross-modal, (3) additive multisensory, (4) nonadditive multisensory, (5) nonresponsive neurons. Units that significantly changed their firing only after unimodal stimulation were classified as unimodal neurons (i.e., tactile stimulation for neurons in S1, visual stimulation for neurons in V1). Units that significantly changed their firing only after cross-modal stimulation were classified as cross-modal neurons (i.e., visual stimulation for neurons in S1, tactile stimulation for neurons in V1). Units that did not significantly modify their firing after any stimulation type were classified as nonresponsive neurons. Units were regarded as multisensory neurons if they either responded to all stimulation types (unimodal, cross-modal, and bimodal) or when the response to the bimodal stimulus was significantly different compared with that to the unimodal stimulus. This bimodal modulation was quantified as previously described (Kayser et al., 2008). First, we determined whether the bimodal response was significantly enhanced or suppressed compared with the unimodal stimulation. This strength of enhancement or suppression was quantified using the enhancement index

$$\mathrm{enhancement}=\frac{bimodal-unimodal}{unimodal+bimodal}\times 100$$

where bimodal and unimodal correspond to the maximal unimodal and bimodal firing response, respectively. This measure informs about the strength of the enhancement or suppression effect. The maximal unimodal response corresponded always to where neural activity was measured (i.e., tactile response for measurements in S1, visual response for measurements in V1). To determine whether this bimodal response modulation was equal to an additive summation of the unimodal stimulations or corresponded to a supra- or subadditive effect, a bootstrapping method was applied in a second step (Stanford et al., 2005; Kayser et al., 2008; Felch et al., 2016). For this, we generated a matrix of the sums of spikes after all possible unimodal trial-by-trial combinations (100 tactile stimulations × 100 visual stimulations). We repeatedly selected 100 samples of this matrix for 10,000 times in a randomized order with replacement. From these 10,000 samples, we created a population mean of the firing rate against which we compared the observed firing rate of each neuron after bimodal stimulation by computing the *z*-score. The deviation from additivity was quantified using the additivity index:

$$\mathrm{additivity}=\frac{bimodal-\left(unimodal+cross-modal\right)}{bimodal+\left(unimodal+cross-modal\right)}\times 100$$

where unimodal, cross-modal, and bimodal reflect the tactile, visual, and visual-tactile (S1) as well as the visual, tactile, and visual-tactile (V1) responses. Neurons that showed a significant effect in the additivity index by deviating from the generated normal distribution (*p* < 0.05) were regarded as nonadditive multisensory. Positive or negative additivity values correspond to supra- or subadditive effects, respectively. In contrast, units were classified as additive multisensory if they showed significant firing changes in response to all types of stimulations but the additivity index did not reach significance.

#### Spike synchrony

Cross-correlation between spike trains in S1 and V1 after bimodal stimulation was used as measure of synchrony and calculated using the Matlab function *xcorr* (5 ms bin size, 3 ms step size, time lag ± 1 s) with V1 firing as reference. The cross-correlation values between S1 and V1 after bimodal visual-tactile stimulation were corrected for spurious coherence by subtracting the cross-correlation values between S1 spike trains after unimodal tactile stimulation and V1 spike trains after unimodal visual stimulation. Unimodal tactile and unimodal visual stimulations were presented at different time points during the stimulation paradigm, and hence should not show any correlation of firing. All PYRs and INs of all classified neuronal groups (unimodal, cross-modal, additive multisensory, nonadditive multisensory) with a significant firing response to stimulation were included in the cross-correlation analysis. Only pairs of neurons with significant cross-correlation values (3.29 SD/99.9 CI threshold) for at least 10 consecutive bins were considered for analysis. A Gaussian smoothing filter was applied to the 1D signal array.

#### Phase coupling analysis

The intercortical phase and strength of locking between the spiking of clustered units and network oscillations was assessed using a previously described algorithm (Siapas et al., 2005; Brockmann et al., 2011). For this, the raw LFP signal was bandpass filtered (4-12, 12-30, and 30-100 Hz) using a third-order Butterworth filter preserving phase information. Subsequently, a Hilbert transform was applied to the filtered signal. If the firing of a neuron is modulated by oscillations within a specific frequency band, then its phase over the oscillatory cycle is not uniformly distributed. Phases of zero referred to the peak and a phase of π/-π referred to the trough of the cycle. The coupling between spikes and network oscillations was tested for significance using the Rayleigh test for nonuniformity. The spike trains were converted into a sequence of unit length vectors oriented by the phase of their corresponding spikes. The value of Rayleigh’s *Z* statistic indicates strength of phase coupling (or degree of nonuniformity) between unit events and field potential and was computed by

$$Z=nR^{2}$$

where R denotes the mean resultant vector (MRV) length of the given phase series. The probability that the null hypothesis of sample uniformity holds is given by

$$P=e^{-z}[1+\frac{2Z-Z^{2}}{4n}-\frac{24Z-132Z^{2}+76Z^{3}-9Z^{4}}{288n^{2}}]$$

For *n* > 50, *>P* = e^-Z^ approximation is adequate (Fisher, 1993). Only neurons that showed a significant degree of phase locking were considered for analyses. Their MRV length (locking strength) as well as their mean direction (preferred phase of locking) were calculated.

The phase locking of spikes to oscillatory activity was confirmed using the pairwise phase consistency (PPC) measure that is independent of the numbers of trials or spikes (Vinck et al., 2010; Tamura et al., 2016). For this, the average pairwise circular distance (D) was calculated as

$$D=\frac{2}{N(N-1)}\sum_{j=1}^{N-1}\sum_{k=(j+1)}^{N}d(\theta j,\theta k)$$

where *D* is the absolute angular distance between two samples, *θj* and *θk* are the phases of LFP samples assigned to contemporaneous spikes, and *N* is the number of spikes. The PPC results from the normalization of D as follows:

$$PPC=\frac{\pi -2D}{\pi }.$$

PPC = 1 indicates complete phase consistency, whereas lack of phase locking leads to PPC = 0. Negative values of PPC correspond to uniformly distributed spikes.

#### Statistics

Statistical analyses were performed using Matlab or IBM SPSS Statistics version 22.0 (IBM). Gaussian distribution of the data were assessed using the Kolmogorov-Smirnov test. Normally distributed data were tested for significant differences (*p < 0.05, **p < 0.01, and ***p < 0.001) using unrelated *t* test. Data that did not follow a Gaussian distribution were tested with Wilcoxon signed-rank test for paired data or with the Mann-Whitney *U* test for nonpaired data. Count data were analyzed with the two proportion *z* test. Nonuniformity of circular data were assessed using the Rayleigh test. Significant differences in the preferred phase of neuronal firing to oscillatory activity were assessed using the nonparametric *circ_cm* test of the Matlab circular statistics toolbox (Berens, 2009). Data are shown as mean ± SEM.

## Results

### Cross-modal stimulation modulates the firing rates of neuronal subpopulations in primary somatosensory and visual cortices

**Figure 1.**
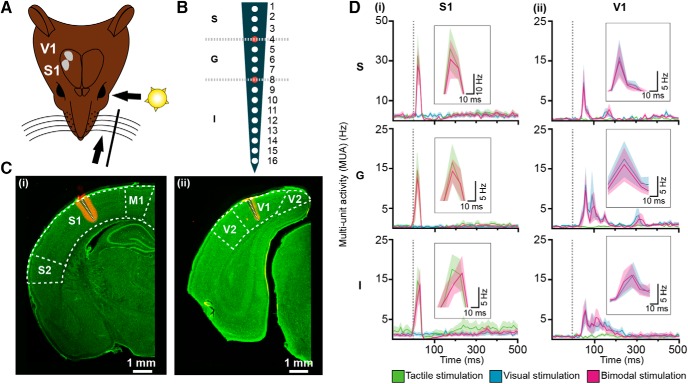
MUA evoked in S1 and V1 by uni- and bimodal stimulation. ***A***, Schematic drawing displaying the protocol for sensory stimulation via whisker deflections and/or light flashes as well as the location of extracellular recordings in S1 and V1 of Brown Norway rats. ***B***, Schematic drawing of a 16-site silicon probe spanning the cortical layers (S = supragranular, G = granular, I = infragranular). Red-filled recording sites at transitions between cortical layers were not considered for analysis. ***C***, (i) Digital photomontage reconstructing the position of all recording sites (white dots) of a Dil-labeled probe in S1. (ii) Same as (i) for V1. ***D***, (i) Line graphs displaying MUA in supragranular (S), granular (G), and infragranular (I) layers after uni- and bimodal stimulation. Stimulus is marked by the gray dotted line. Insets show the peaks of MUA after stimulation at higher magnification. (ii) Same as (i) for V1. Note that unimodal visual or unimodal tactile stimulation did not evoke responses in S1 or V1, respectively.

To elucidate the cellular mechanisms of multisensory integration, we firstly assessed the population and individual firing rates after uni- and bimodal stimulation in supragranular (S), granular (G), and infragranular (I) layers of S1 and V1 (Fig. 1*A*,*C*). For this, we examined the MUA recorded at multiple sites over the cortical depth (Fig. 1*B*) in lightly urethane-anesthetized Brown Norway rats. The good visual acuity of pigmented Brown Norway rats makes them well suited for testing visual-somatosensory processing. By conducting the entire investigation under sleep-like conditions mimicked by urethane anesthesia (Clement et al., 2008; Bitzenhofer et al., 2015), we avoided the interference with spontaneous whisking and the impact of alert state, which both modulate cross-modal interactions. However, the processing mechanisms identified here may differ from those taking place in awake state, since sleep-like conditions have been shown to increase single unit response variability to bimodal stimulation (Populin, 2005).

In S1, contralateral whisker stimulation led to a strong increase of MUA peaking at 32.20 ± 10.64 Hz in S layer, 14.53 ± 4.39 Hz in G layer, and 14.48 ± 3.69 Hz in I layer after 14.7, 12.29, and 15.05 ms from stimulus onset, respectively. This increase was followed by a nonsignificant decrease and later by a long-lasting low-magnitude augmentation of firing when compared with the baseline (Fig. 1*D*, i). In V1, contralateral light stimulation caused broad peaks of augmented MUA after ∼70.84 ms in all cortical layers (Fig. 1*D*, ii). Bimodal stimulation similarly changed the firing rates in S1 and V1. Despite a small decrease, no significant differences were detected when compared with the spiking dynamics in S1 (S: 27.59 ± 9.71 Hz, *p* ^a^ = 0.31; G: 12.37 ± 3.02 Hz, *p* ^b^ = 0.16; I: 13.58 ± 4.36 Hz, *p* ^c^ = 0.4; Fig. 1*D*, i) and V1 (S: 8.5 ± 4.05 Hz, *p* ^d^ = 0.5; G: 9.60 ± 4.24 Hz, *p* ^e^ = 0.19; I: 7.17 ± 2.93 Hz, *p* ^f^ = 0.3; Fig. 1*D*, ii) after unimodal stimulation. Thus, population activity in primary sensory cortices was not significantly changed by bimodal visual-tactile stimulation when compared with unimodal modality-specific stimulation.

**Figure 2.**
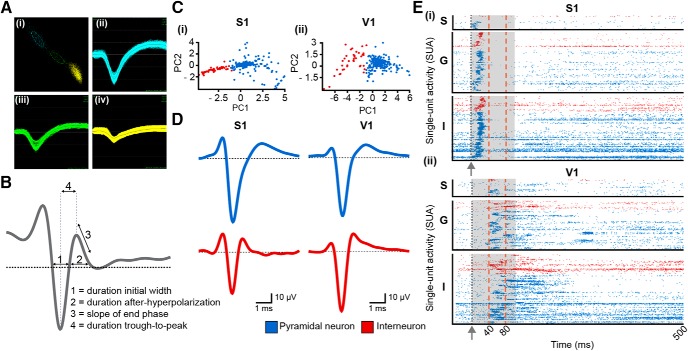
Classification of single units according to their electrophysiological phenotype**. *A***, Example of clustered action potential waveforms (i) of three neurons recorded in S1 granular layers (ii–iv). ***B***, Schematic drawing of a spike wave form and of features that were used for classification of units into PYRs or INs. ***C***, (i) Two-dimensional scatter plot of feature vectors into the PC space spanned by the first two PCs with k-means assignment of class membership (blue = PYR, red = IN) of S1 units. (ii) Same as (i) for V1 units. ***D***, Example waveforms of a classified PYR (top) and IN (bottom) in G layer of S1 and V1. ***E***, (i) Raster plot depicting spike trains recorded from PYRs (blue) and INs (red) in supragranular (S), granular (G), and infragranular (I) layers of S1 after bimodal stimulation (gray arrow and dotted line). Each line corresponds to one trial and each dot to one spike. The temporal organization of spiking patterns was used for identifying time windows for further analyses [before stimulus, early stimulus-induced response: 0-40 ms (S1), 0-80 ms (V1); late stimulus-induced response: 80-500 ms after stimulus (S1 and V1)]. Insets (gray boxes) correspond to the time intervals that were used for SUA quantification leading the classification of neurons into unimodal, cross-modal, additive multisensory, nonadditive multisensory, and unresponsive. (ii) Same as (i) for V1.

To investigate whether bimodal stimulation influences the firing of individual neurons in S1 and V1, we performed cluster analysis of MUA followed by classification of units into PYRs or INs according to four major spike waveform features (see Materials and Methods; Fig. 2*A-D*). By these means, 81% of S1 neurons (212 out of 262 neurons) and 83% of V1 neurons (204 out of 245) were identified as putatively PYRs. The low fraction of spiking shapes assigned to INs (S1 over all layers: 15.83 ± 0.05%, V1 over all layers: 14.29 ± 0.04%) is in agreement with previous anatomic and functional studies (Somogyi et al., 1998; Gupta et al., 2000; Markram et al., 2004; Rudy et al., 2011). The recorded PYRs and INs were similarly distributed over S1 and V1 layers (S1: S layer, 10 PYRs and 1 INs, V1: S layer, 13 PYRs and 1 INs; S1: G layer, 93 PYRs and 32 INs; V1: G layer, 78 PYRs and 22 INs; S1: I layer, 109 PYRs and 16 INs, V1: I layer, 113 PYRs and 18 INs). In line with MUA dynamics over time, the temporal organization of pyramidal and interneuronal firing in S1 after whisker deflection differed from the firing induced by light stimulation in V1. While in S1 the firing of both PYRs and INs increased during the first 40 ms after stimulus and significantly decreased during the subsequent 40 ms, in V1, the pyramidal and interneuronal discharge firstly increased for 40-80 ms after stimulus and remained constant for ∼200 ms thereafter (Fig. 2*E*). These temporal differences in unisensory responses are in line with previous results (Wang et al., 2008; Ghoshal et al., 2011) and reflect preprocessing differences along the anatomic pathways (Petersen, 2007; Cruz-Martín et al., 2014). As expected, for both S1 and V1, the shortest response onset was detected in G layers.

**Figure 3.**
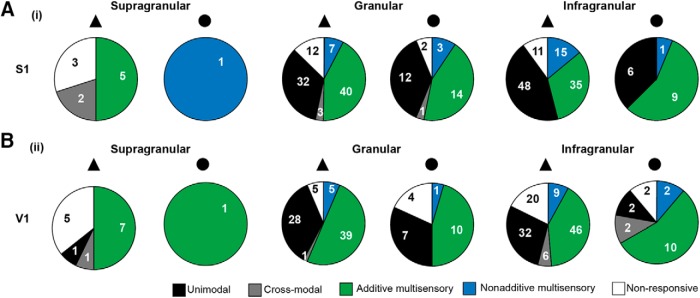
Classification of neurons in S1 and V1 according to their spiking response to uni- and bimodal stimulation. ***A***, Pie charts quantifying the distribution of unimodal, cross-modal, additive multisensory, nonadditive multisensory, and unresponsive PYRs (triangle) and INs (circles) in supragranular, granular, and infragranular layers of S1. Numbers inside the pie chart indicate the total count of neurons in that class. ***B***, Same as ***A*** for V1.

Previous studies showed that neurons in putatively unisensory primary cortices heterogeneously respond to uni- and bimodal stimulation (Wallace et al., 2004; Meredith and Allman, 2015). Correspondingly, we analyzed in detail the firing patterns of individual neurons in S1 and V1 after tactile, visual, and bimodal (i.e., visual-tactile) stimulation (Fig. 3). According to the responsiveness to these three types of stimuli, the neurons were classified in five groups: (1) unimodal neurons (i.e., responsive to unimodal stimulation only), (2) cross-modal neurons (i.e., responsive to unimodal stimulation of the opponent modality), (3) additive multisensory neurons (responsive to unimodal, cross-modal, and bimodal stimulation with no significant difference between unimodal and bimodal stimulation, or responsive to unimodal, cross-modal, and bimodal stimulation where the response to bimodal stimulation significantly differed from unimodal stimulation but was not supra- or subadditive when compared with the arithmetic sum of unimodal and cross-modal stimulation responses), (4) nonadditive multisensory neurons (responsive to unimodal, cross-modal, and bimodal stimulation; the response to bimodal stimulation significantly differed from unimodal stimulation, being supra- or subadditive when compared with the arithmetic sum of unimodal and cross-modal stimulation responses), and (5) nonresponsive neurons (i.e., responsive to none of the stimulations). Multisensory additive neurons had the highest prevalence across layers in S1 and V1 (S1–S: 50% PYRs, 0% INs; G: 43% PYRs, 44% INs; I: 32% PYRs, 56% INs; V1–S: 47% PYRs, 100% INs; G: 50% PYRs, 45% INs; I: 41% PYRs, 56% INs). In most of the cases, they displayed a significant, but similar firing change in response to unimodal, cross-modal, and bimodal stimulation. These neurons exert their multisensory effects at subthreshold level. Only a few neurons in the multisensory additive group displayed a significant difference between unimodal and bimodal stimulation (Fig. 3*A*,*B*). These neurons were distributed only across G and I layers (S1–G: 1 PYR, 3 IN; I: 7 PYR, 9 IN; V1–G: 1 PYR, 0 IN; I: 3 PYR, 0 IN). The strength of the firing change of these neurons monitored by the average percentage increase or decrease of firing that is attributed to the concurrent presentation of visual-tactile stimulations (*enhancement*) varied between cell type and layer (S1–G: PYR 80%, IN 66%; I: PYR 42%, IN 36%; V1–G: PYR 60%, I: PYR 48%). The second most frequent neuronal class was classified as unimodal neurons and was found across all layers in V1 (S: 7% PYR, 0% IN; G: 36% PYR, 32% IN; I: 28% PYR, 11% IN) and in the G (34% PYR, 38% IN) and I layers (44% PYR, 38% IN) of S1. The third largest neuronal class comprised the nonadditive multisensory neurons. They could be found in all layers of S1 (S: 0% PYR, 100% IN; G: 7% PYR, 9% IN; I: 14% PYR, 6% IN) and in the G (6% PYR, 5% IN) and I layers (8% PYR, 11% IN) of V1. The median deviation from additivity of nonadditive neurons differed between cell type and layers (S1–G: PYR -70%, IN -90%; I: PYR -61%, IN -65%; V1–G: PYR -70%, IN -64%; I: PYR -58%, IN -47%).

**Figure 4.**
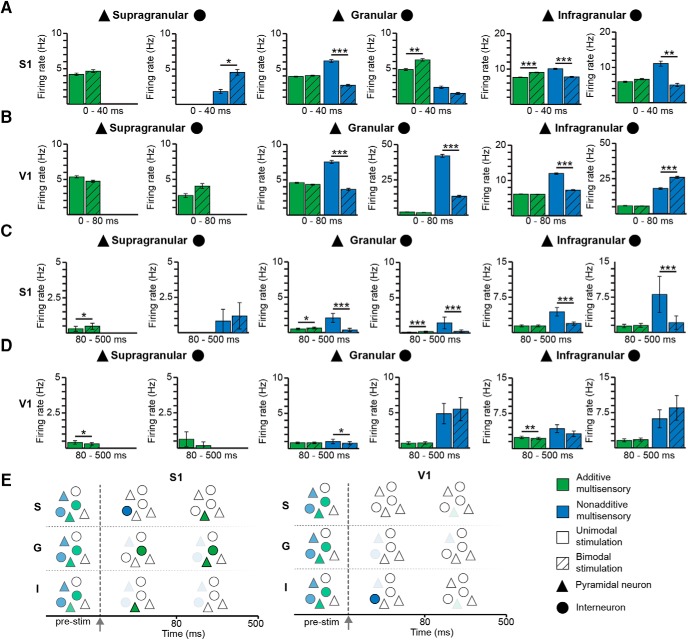
Modulation of firing rate by uni- and bimodal stimulation. ***A***, Bar diagrams showing the firing rate of additive (green) and nonadditive (blue) multisensory PYRs (triangle) and INs (circle) in supragranular, granular, and infragranular layers of S1 during the first 40 ms after unimodal (solid bar) and bimodal (striped bar) stimulus. ***B***, Same as ***A*** for V1. ***C***, Bar diagrams showing the firing rate of additive (green) and nonadditive (blue) multisensory PYRs (triangle) and INs (circle) in supragranular, granular, and infragranular layers of S1 during 80-500 ms after unimodal (solid bar) and bimodal (striped bar) stimulus. ***D***, Same as ***C*** for V1. ***E***, Schematic diagram displaying the modulation of PYRs and INs in supragranular, granular, and infragranular layers by bimodal stimuli. Lower transparency corresponds to rate increase, whereas higher transparency codes rate decrease. Bimodal stimulation is marked by gray arrow and dotted line.

To examine the modulation of firing rates of additive multisensory and nonadditive multisensory neurons during cross-modal processing in more detail, we compared SUA after unimodal and bimodal stimulation (Fig. 4). In S1, the firing of nonadditive multisensory INs in S layer (*p* ^g^ = 0.03) and to a lower extent of the additive multisensory neurons in G layer (*p* ^h^ < 0.01) significantly increased after bimodal stimulation (Fig. 4*A*; Table 1). In contrast, PYRs in G layer, which were classified as nonadditive multisensory neurons decreased their firing after bimodal stimulation (*p* ^i^ < 0.001; Fig. 4*A*; Table 1). The firing rates of neurons located in I layer were similarly modified after uni- versus bimodal stimulation. In the G layer of V1, the firing of nonadditive multisensory neurons was significantly decreased after bimodal stimulation, the most prominent effects being detected for INs (*p* ^j^ < 0.001) (Fig. 4*B*; Table 1). In I layer of V1, PYRs assigned to the nonadditive multisensory neurons decreased their firing (*p* ^k^ < 0.001), whereas the INs belonging to the same group significantly augmented their firing (*p* ^l^ < 0.001; Fig. 4*B*; Table 1). These results indicate that, even if bimodal stimulation did not significantly modulate population firing rates monitored by MUA, it changes the firing rate of discrete groups of individual multisensory neurons. In S1, the most prominent changes were detected in S and G layer with a strong attenuation of pyramidal firing and an augmentation of interneuronal firing. A similar response pattern was detected in V1, yet neurons across all layers responded within the first 80 ms.

**Table 1.** Firing rate of cortical neurons across layers

Area	Cell type	Time	Layer											
			Supragranular				Granular				Infragranular			
			PYR (Hz)		IN (Hz)		PYR (Hz)		IN (Hz)		PYR (Hz)		IN (Hz)	
			Uni	Bi	Uni	Bi	Uni	Bi	Uni	Bi	Uni	Bi	Uni	Bi
S1	Additive multisensory	I	4.20 ± 0.79	4.65 ± 0.68(n.s.)			3.85 ± 0.06	3.98 ± 0.07(n.s.)	4.84 ± 0.14	6.23 ± 0.16(**)	7.60 ± 0.11	9.08 ± 0.11(***)	5.92 ± 0.17	6.64 ± 0.19(n.s.)
	Nonadditive multisensory	I			1.75 ± 0.29	4.5 ± 0.39(*)	6.04 ± 0.19	2.61 ± 0.13(***)	2.33 ± 0.17	1.50 ± 0.14(n.s.)	10.02 ± 0.16	7.63 ± 0.14(***)	11.0 ± 0.72	5.0 ± 0.47(**)
	Additive multisensory	II	0.26 ± 0.79	0.45 ± 0.59(**)			0.25 ± 0.06	0.30 ± 0.07(*)	0.09 ± 0.05	0.16 ± 0.07(***)	1.38 ± 0.21	1.41 ± 0.21(n.s.)	0.88 ± 0.25	0.96 ± 0.28(n.s.)
	Nonadditive multisensory	II			0.83 ± 0.83	1.20 ± 0.93(n.s.)	1.04 ± 0.32	0.19 ± 0.11(***)	1.43 ± 0.83	0.24 ± 0.19(***)	4.43 ± 0.88	1.91 ± 0.34(***)	5.30 ± 2.53	1.35 ± 0.96(***)
V1	Additive multisensory	I	5.29 ± 0.15	4.67 ± 0.15(n.s.)	2.66 ± 0.27	4.00 ± 0.36(n.s.)	4.51 ± 0.06	4.30 ± 0.06(n.s.)	2.10 ± 0.08	1.70 ± 0.08(n.s.)	6.12 ± 0.07	6.01 ± 0.06(n.s.)	5.77 ± 0.14	1.59 ± 0.13(n.s.)
	Nonadditive multisensory	I					7.47 ± 0.21	3.60 ± 0.15(***)	42.0 ± 1.04	13.33 ± 0.65(***)	11.85 ± 0.20	7.22 ± 0.15(***)	17.67 ± 0.56	25.83 ± 0.72(***)
	Additive multisensory	II	0.79 ± 0.24	0.59 ± 0.20(*)	0.60 ± 0.51	0.18 ± 0.26(**)	0.84 ± 0.12	0.84 ± 0.11(n.s.)	0.70 ± 0.18	0.68 ± 0.18(n.s.)	1.53 ± 0.16	1.41 ± 0.16(**)	1.59 ± 0.31	1.73 ± 0.35(n.s.)
	Nonadditive multisensory	II					0.97 ± 0.32	0.72 ± 0.24(n.s.)	4.85 ± 1.46	5.50 ± 1.64(n.s.)	2.80 ± 0.55	2.07 ± 0.39(***)	6.15 ± 1.91	8.48 ± 2.63(***)

Firing rate of additive and nonadditive multisensory neurons in S1 and V1. Values are displayed as mean ± SEM for both unimodal (uni) and bimodal (bi) stimulation. Significant differences between stimulations are indicated in parentheses (n.s., not significant; *p < 0.05, **p < 0.01, ***p < 0.001, Wilcoxon signed-rank test) for the early and late poststimulus intervals in which firing was assessed [I = 0-40 ms (S1), 40-80 ms (V1); II = 80-500 ms].

While the categorization of neuronal subpopulations into the previously described five groups was made according to their immediate response to uni- versus bimodal stimulation, additive as well as nonadditive multisensory neurons showed stimulus-induced changes of firing at later time points (i.e., 80-500 ms) as well (Fig. 4*C*,*D*; Table 1). These late effects of variable magnitude across areas, layers, and cell type (PYRs vs INs) most likely had a polysynaptic origin, and consisted in most cases of a decrease of neuronal firing after bimodal stimulation when compared with unimodal stimulation.

Taken together, these results reveal complex patterns of firing rate modulation by visual-tactile stimulation in a relatively small fraction of the many multisensory neurons in primary sensory cortices (Fig. 4*E*). Overall, the INs increased their firing rate, whereas PYRs mainly decreased their firing rate within the first 80 ms after stimulus. Thus, broad depression of stimulus-induced excitatory neuronal activity after bimodal stimulation is accompanied by enhanced firing of a sparse number of additive and nonadditive multisensory INs.

### Cross-modal stimulation modulates the firing latencies and phase-coupling in area- and cell type-specific manner

Not only the firing rate but also the timing of neuronal discharges has been proposed to contribute to multisensory processing and improve the behavioral performance (Bizley et al., 2007; Rowland et al., 2007; Chabrol et al., 2015). On the one hand, bimodal stimulation may modify the firing latency and, hence, the delay between sensory- and motor-related activation with consequences for behavioral performance. On the other hand, bimodal stimulation may alter the locking time and strength of neuronal firing to the phase of network oscillations.

**Figure 5.**
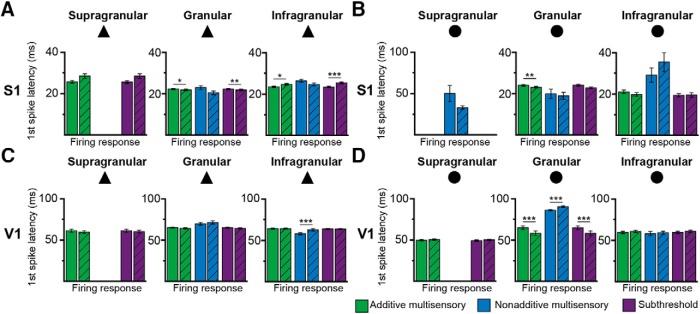
Modulation of first spike latency by uni- and bimodal stimulation. ***A***, Bar diagram displaying the mean latency of the first spike of additive multisensory (green), nonadditive multisensory (blue), and subthreshold (purple) PYRs (triangles) in supragranular, granular, and infragranular layers after unimodal (solid) and bimodal (striped) stimulation. ***B***, Same as ***A*** for INs (circle) in S1. ***C***, Same as ***A*** for PYRs in V1. ***D***, Same as ***B*** for INs in V1.

To decide which temporal coding strategy serves for the processing of visual-tactile stimuli, we firstly compared the spiking latencies of cells identified as PYRs and INs in S layer (*n* = 15 cells, *n* = 2 cells), G layer (*n* = 140 cells, *n* = 45 cells), and I layer (*n* = 165 cells, *n* = 31 cells) of S1 and V1, respectively, after bimodal and unimodal stimulation. To avoid the influence of firing rate on the measured first spike latency, we separately analyzed the spiking onset of additive multisensory neurons that only showed subthreshold multisensory responses (i.e., significantly responded to unimodal tactile, unimodal visual, and bimodal stimulation but with no significant difference between the unimodal and bimodal stimulation). In G layer of S1, the latency of the first spike of subthreshold multisensory PYRs significantly decreased after bimodal stimulation when compared with unimodal stimulation (*p* ^m^ < 0.01) (Fig. 5*A*; Table 2). In contrast, the latency of subthreshold pyramidal firing in I layers increased after bimodal stimulation (*p* ^n^ < 0.001; Fig. 5*A*; Table 2 and 3). In V1 G layers, subthreshold INs responded faster after bimodal stimulation when compared with unimodal stimulation (*p*° < 0.001; Fig. 5*D*; Table 2). Over all layers of S1 and V1, INs (S1: 20.45 ± 1.86; V1: 56.8 ± 2.86) and PYRs (S1: 23.45 ± 1.03; V1: 57.58 ± 3.32) did not significantly differ in their firing onset (*p* ^p^ = 0.52). The similar response timing of PYRs and INs is in line with previous studies showing that thalamic relay cells target both excitatory and inhibitory L4 neurons (Kloc and Maffei, 2014; Yu et al., 2016). These results indicate that visual-tactile stimulation modulates the onset of neuronal firing in primary sensory cortices.

**Table 2.** First-spike latencies of cortical neurons across layers

		Additive multisensory		Nonadditive multisensory		Subthreshold	
		Unimodal	Bimodal	Unimodal	Bimodal	Unimodal	Bimodal
				Supragranular			
S1	PYR	25.65 ± 0.62	28.55 ± 1.08 (n.s.)			25.65 ± 0.63	28.55 ± 1.08 (n.s.)
	IN			50.22 ± 9.71	32.88 ± 9.71 (n.s.)		
V1	PYR	61.30 ± 1.68	59.81 ± 1.62 (n.s.)			61.30 ± 1.68	59.81 ± 1.62 (n.s.)
	IN	49.43 ± 0.80	49.98 ± 0.73 (n.s.)			49.43 ± 0.80	49.98 ± 0.73 (n.s.)
				Granular			
S1	PYR	22.33 ± 0.29	21.92 ± 0.40 (*)	22.98 ± 0.92	20.46 ± 0.92 (n.s)	22.32 ± 0.29	21.94 ± 0.40 (**)
	IN	24.12 ± 0.41	23.10 ± 0.42 (**)	19.92 ± 2.30	19.03 ± 2.30 (n.s.)	24.07 ± 0.43	22.92 ± 0.50 (n.s.)
V1	PYR	64.96 ± 0.74	64.04 ± 0.72 (n.s.)	69.53 ± 1.64	71-36 ± 1.64 (n.s.)	64.87 ± 0.73	63.92 ± 0.72 (n.s.)
	IN	64.75 ± 2.26	57.81 ± 2.87 (***)	86.14 ± 0.53	90.45 ± 0.53 (***)	64.75 ± 2.26	57.81 ± 2.87 (***)
				Infragranular			
S1	PYR	23.56 ± 0.37	24.75 ± 0.33 (*)	26.43 ± 0.75	24.75 ± 0.75 (n.s.)	23.49 ± 0.41	25.41 ± 0.42 (***)
	IN	20.94 ± 0.90	19.65 ± 0.90 (n.s.)	28.90 ± 3.49	35.47 ± 3.49 (n.s.)	19.41 ± 0.74	19.55 ± 1.12 (n.s.)
V1	PYR	63.96 ± 0.68	63.85 ± 0.68 (n.s.)	57.98 ± 1.29	62.87 ± 1.29 (***)	63.86 ± 0.69	63.69 ± 0.71 (n.s.)
	IN	59.74 ± 1.44	60.92 ± 1.34 (n.s.)	58.37 ± 2.51	59.37 ± 2.51 (n.s.)	59.74 ± 1.44	60.92 ± 1.34 (n.s.)

Stimulus induced first spike latencies of neurons in S1 and V1. Values are displayed as mean ± SEM for both unimodal (uni) and bimodal (bi) stimulations. Significant differences between stimulations are indicated in parentheses (n.s., not significant; *p < 0.05, **p < 0.01, ***p < 0.001, Wilcoxon signed-rank test).

**Table 3.** Statistical Table

	Data structure	Type of test	Power
a.	Non-normal data	Wilcoxon signed-rank test	*p* = 0.31
b.	Non-normal data	Wilcoxon signed-rank test	*p* = 0.16
c.	Non-normal data	Wilcoxon signed-rank test	*p* = 0.40
d.	Non-normal data	Wilcoxon signed-rank test	*p* = 0.50
e.	Non-normal data	Wilcoxon signed-rank test	*p* = 0.19
f.	Non-normal data	Wilcoxon signed-rank test	*p* = 0.30
g.	Non-normal data	Wilcoxon signed-rank test	*p* = 0.03
h.	Non-normal data	Wilcoxon signed-rank test	*p* < 0.01
i.	Non-normal data	Wilcoxon signed-rank test	*p* < 0.001
j.	Non-normal data	Wilcoxon signed-rank test	*p* < 0.001
k.	Non-normal data	Wilcoxon signed-rank test	*p* < 0.001
l.	Non-normal data	Wilcoxon signed-rank test	*p* < 0.001
m.	Non-normal data	Wilcoxon signed-rank test	*p* < 0.01
n.	Non-normal data	Wilcoxon signed-rank test	*p* < 0.001
o.	Non-normal data	Wilcoxon signed-rank test	*p* < 0.001
p.	Non-normal data	Wilcoxon signed-rank test	*p* = 0.52
q.	Circular data	Circ_mean (circular statistics toolbox, computes mean direction)	*p* < 0.05
r.	Count data	Two proportion *z* test	*p* < 0.001
s.	Count data	Two proportion *z* test	*p* < 0.05
t.	Count data	Two proportion *z* test	*p* < 0.001
u.	Count data	Two proportion *z* test	*p* < 0.01
v.	Circular data	Circ_r (circular statistics toolbox, computes MRV length)	*p* < 0.001
w.	Circular data	Circ_r (circular statistics toolbox, computes MRV length)	*p* < 0.001
x.	Circular data	Circ_r (circular statistics toolbox, computes MRV length)	*p* < 0.001
y.	Circular data	Circ_r (circular statistics toolbox, computes MRV length)	*p* < 0.001
z.	Count data	Two proportion *z* test	*p* < 0.001
aa.	Count data	Two proportion *z* test	*p* < 0.001
bb.	Count data	Two proportion *z* test	*p* < 0.001
cc.	Count data	Two proportion *z* test	*p* < 0.01
dd.	Circular data	Circ_r (circular statistics toolbox, computes MRV length)	*p* < 0.001
ee.	Circular data	Circ_r (circular statistics toolbox, computes MRV length)	*p* < 0.01
ff.	Normal data	99% CI threshold	99% threshold in granular layer: a cross-correlation coefficient larger than 0.0013 (baseline) (median of individual spike pair values 0.012, 25 percentile: 0.009, 75 percentile: 0.03)99% threshold in infragranular: a cross-correlation coefficient larger than 0.0045 (baseline)(median of spike pair values 0.034, 25 percentile: 0.023, 75 percentile: 0.058)

**Figure 6.**
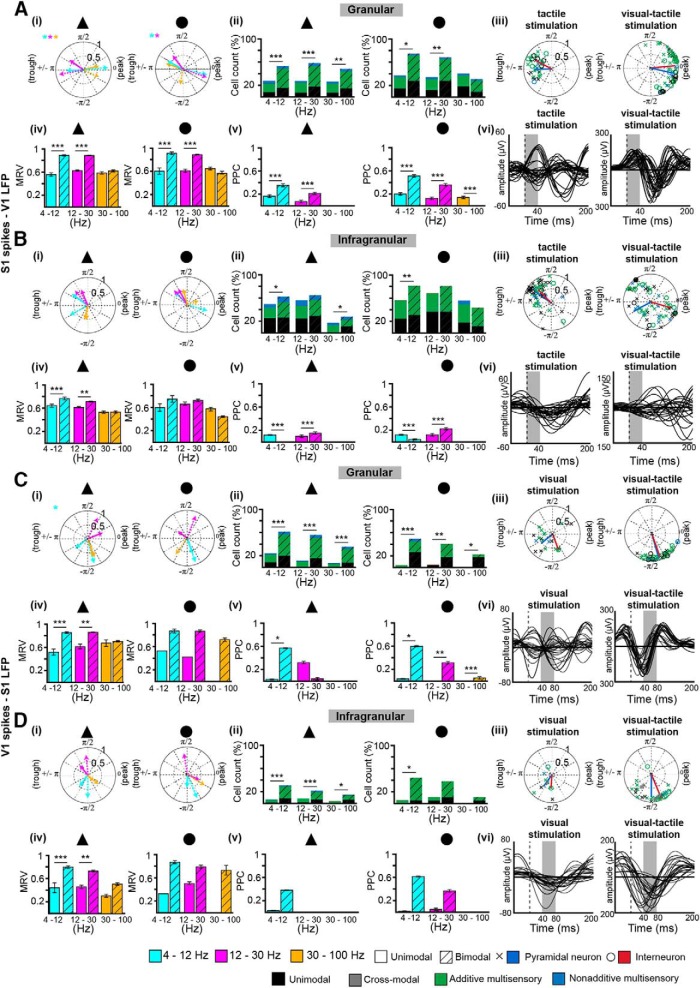
Modulation of spike-LFP coupling by uni- and bimodal stimulation. ***A***, (i) Polar plots depicting the MRV of S1 phase-locked PYRs (triangle) and INs (circle) to 4-12 Hz (cyan), 12-30 Hz (magenta), and 30-100 Hz (orange) V1 network oscillations after unimodal (solid line) and bimodal stimulation (dashed line). Significant phase differences between uni- and bimodal conditions are indicated by * (color coded to the frequency range of V1 oscillations). (ii) Bar diagrams displaying the fraction of PYRs (triangles) and INs (circles) significantly phase-locked to 4-12, 12-30, and 30-100 Hz oscillations before (left) and after (right) bimodal stimulation of the categorized neurons (black = unimodal, gray = cross-modal, green = additive multisensory, blue = nonadditive multisensory). (iii) Circle plot depicting the phase locking of unimodal (black), cross-modal (gray), additive multisensory (green), and nonadditive multisensory (blue) PYRs (triangle) and INs (circle) in the S1 after unimodal (right) and bimodal stimulation (left) to V1 theta oscillations. (iv) Bar diagram displaying the MRV of S1 PYRs (triangle) and INs (circle) locked to V1 4-12 Hz (cyan), 12-30 Hz (magenta), and 30-100 Hz (orange) network oscillations. (v) Same as (iv) for the PPC measure. (vi) Diagram depicting the V1 theta oscillations onto which the S1 neurons depicted in (iii) are locked to. ***B***, Same as ***A*** for S1 infragranular neurons phase-locked to V1 infragranular oscillations. ***C***, Same as ***A*** for V1 granular neurons phase-locked to S1 granular oscillations. ***D***, Same as ***C*** for V1 infragranular neurons phase-locked to S1 infragranular oscillations.

Second, we investigated whether bimodal stimulation affects the temporal coupling between individual neuronal spiking and network oscillations. For this, we analyzed the locking of spikes to the phase of LFP oscillations recorded in the ipsilateral V1 and S1, respectively, by calculating the MRV and confirming the results by PPC analysis (Fig. 6*A-D*). Similarly to the previous studies (Harris et al., 2016), the low number of clustered neurons in S layers of S1 and V1 precluded reliable assessment of their phase locking, and therefore, only spiking from neurons in G and I layers significantly locked to V1 4 -100 Hz network oscillations were considered. The proportion of phase-locked neurons tended to decrease with increasing frequency from 4-12 Hz (S1–PYR unimodal: 40.59%, bimodal: 58.42%; IN unimodal: 43.75%, bimodal: 77.08%; V1–PYR unimodal: 10.47%, bimodal: 42.93%; IN unimodal: 5.00%, bimodal: 47.50%) to 30-100 Hz (S1–PYR unimodal: 27%, bimodal: 58%; IN unimodal: 41%, bimodal: 28%; V1–PYR unimodal: 6%, bimodal: 34%; IN unimodal: 0%, bimodal: 23%; Fig. 6*A*,*C*, ii). Similar effects were found for I layers (Fig. 6*B*,*D*, ii). In S1, the responses of PYRs and INs to bimodal stimulation occurred around the peak of oscillatory theta cycle, whereas after unimodal stimulation, they were concentrated at the trough (*p* ^q^ < 0.05; Fig. 6*A*, i and iii). In addition, bimodal stimulation augmented the number of phase-locked PYRs and INs in G layer (theta–PYR: from 28% to 54%, *p* ^r^ < 0.001, IN: from 38% to 75%, *p* ^s^ < 0.05; beta–PYR: from 27% to 57%, *p* ^t^ < 0.001, IN: from 34% to 69%, *p* ^u^ < 0.01; Fig. 6*A*, ii). Correspondingly, the magnitude of the MRV significantly increased for both cell types for theta (*p* ^v^ < 0.001, *p* ^w^ < 0.001) as well as beta oscillations (*p* ^x^ < 0.001, *p* ^y^ < 0.001; Fig. 6*A*, iv). A higher number of phase-locked PYRs and INs after bimodal stimulation was additionally detected in I layer (theta–PYR: from 49% to 62%, IN: from 56% to 81%, beta–PYR: from 56% to 65%, IN: from 69% to 82%), yet their firing was less precisely timed by the theta–beta oscillatory cycle and correspondingly, the magnitude of the MRV was lower when compared with that calculated for G layer (Fig. 6*B*, i-iv). In V1, the phase locking of PYRs and INs to theta–beta oscillations significantly augmented after bimodal stimulation, both the number of phase-locked cells (theta: PYR *p* ^z^ < 0.001, IN *p* ^aa^ <0.001, beta: PYR *p* ^bb^ < 0.001, IN *p* ^cc^ < 0.01) and the strength of MRV for PYRs being increased (theta: *p* ^dd^ < 0.001, beta: *p* ^ee^ < 0.01; Fig. 6*C*, i-iv, *D*, i-iv). The bimodal induced strengthening of phase locking between spikes and theta–beta phase was confirmed by PPC analysis (Fig. 6*A*, v, through *D*, v). While the augmentation of spike-LFP synchrony may result from the previously reported phase reset of network oscillations, modulation of spike timing occurs also in the absence of such phase reset and is dependent on LFP frequency and neuronal type. These data indicate that visual-tactile stimulation modulates not only the rate but also the timing of pyramidal and interneuronal firing in primary sensory cortices.

### A subset of glutamatergic neurons establishes direct bi-directional connections between S1 and V1

**Figure 7.**
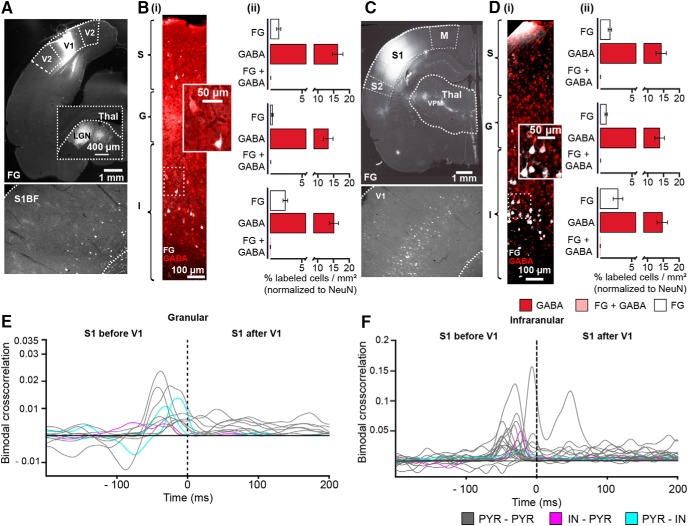
Anatomic and functional characterization of direct S1–V1 connectivity. ***A***, Fluorescence microscopy image displaying the injection site of the retrograde tracer FG covering all V1 layers in a 50-µm-thick cortical slice (top) and retrogradely labeled neurons in S1 (bottom). ***B***, (i) Photograph depicting retrogradely labeled neurons over S1 layers (S, G, I) after FG injection into V1 when costained against GABA in a 50-µm-thick cortical slice. Inset, a GABA- and FG-positive (red) neuron and a GABA-negative FG-positive neuron (white). (ii) Bar diagram displaying the fraction of FG-, GABA-, and FG + GABA-positive neurons in S1 after FG injection in V1. ***C***, Same as ***A*** for injection in S1 and retrogradely labeled neurons in V1. ***D***, Same as ***B*** for V1. ***E***, Line plot displaying the cross-correlation of simultaneously recorded spike trains in the granular layer of S1 and V1 after bimodal stimulation. The cross-correlation after unimodal stimulation was subtracted to correct for spurious coupling. Gray lines correspond to PYR–PYR correlation, whereas cyan and magenta lines indicate PYR–IN and IN–PYR coupling, respectively. ***F***, Same as ***E*** for infragranular layers.

To elucidate the anatomic substrate of visual-tactile processing, we assessed the patterns of direct connectivity between S1 and V1. We previously showed that corticocortical axonal projections may account for cross-modal phase reset of network oscillations (Sieben et al., 2013). Here, we investigate the role of corticocortical connections for the cross-modal modulation of neuronal firing by quantifying the layer- and cell type-specific distribution of projections between S1 and V1. For this, we injected small amounts of the retrograde tracer FG, which has high resistance to fading (Schmued and Fallon, 1986), into the S1 (*n* = 7 rats) or V1 (*n* = 7 rats) taking special attention that the tracer covered all layers without exceeding the cortical area (Fig. 7*A*,*C*). The spatial confinement of injection was verified by back labeling in the corresponding first-order thalamic nuclei of S1 (ventral posteromedial nucleus) and V1 (lateral geniculate nucleus), respectively. Confirming our previous results, bright fluorescence back labeling of parent cell bodies feedforwardly projecting to the S1 barrel field or to V1 were detected when FG was injected into V1 or S1, respectively. We quantified in detail the layer distribution of neurons in one primary sensory area that directly project to the other one. Related to the density of cells positive for the neuronal marker NeuN, a small fraction of neurons contributes to corticocortical coupling. In S1, the highest density was detected in the S layer (1.28 ± 0.33%) and I layer (2.36 ± 0.37%), whereas only 0.3 ± 0.13% of neurons in G layer were retrogradely stained (Fig. 7*A*,*B*). In V1, the distribution was similar with the highest density of FG-positive neurons in S layer (1.53 ± 0.27%) and I layer (2.81 ± 0.76%; Fig. 7*C*,*D*).

To decide on the neurochemical identity of cortico-cortically projecting neurons, GABA staining was performed (Fig. 7*B*,*D*). In line with previous data (Fitzpatrick et al., 1987; Beaulieu, 1993; He et al., 2016), the density of GABA-positive neurons was similar in S1 and V1 and no layer-specific differences could be detected (S1: S layer 16.35 ± 1.66%, G layer 13.30 ± 1.54%, I layer 15.17 ± 1.46%; V1: S layer 14.25 ± 1.63%; G layer 13.79 ± 1.54%; I layer 14.68 ± 1.64%). Very few GABA-positive neurons were back-labeled for FG in I layer of S1 (0.12 ± 0.03%) and I layer of V1 (0.1 ± 0.04%), demonstrating sparse reciprocal GABAergic connections between primary sensory cortices in deep cortical layers.

To uncover the contribution of the sparse cortico-cortical projections for the timing of neuronal firing in S1 and V1, we calculated the coupling strength and delay between spike trains in one cortical area in relationship to the other area after bimodal stimulation (Fig. 7*E*,*F*). The low number and firing rate of clustered units simultaneously recorded in S layers of S1 and V1 precluded the analysis of their temporal spike correlations. Cross-correlation analysis for spike trains recorded in G layers of S1 and V1 identified significantly correlated trains^ff^, yet their number was very low (G: 12 out of 492, I: 24 out of 552). The analysis of cell-type specificity of spike train coupling equally confirmed the anatomic data. The majority of significantly temporally correlated neurons were PYRs projecting onto PYRs (G layer: 75%, I layer 92% of correlated pairs).

The delay between spike trains simultaneously recorded in both areas after bimodal stimulation gave first insights into the directionality of neuronal interactions between S1 and V1 during multisensory processing. At the level of G layers, 82% of PYRs in S1 fired 12.5 ± 0.82 ms before V1 neurons, whereas only 2 out of 11 PYRs fired shortly (6 ± 2.82 ms) after V1 neurons. Similarly, at the level of I layer, the firing of the majority of S1 neurons (22 out of 24) preceded V1 discharges. Thus, bimodal stimulation leads few S1 neurons to drive the firing of intercortically connected V1 neurons.

Taken together, these results indicate that processing of visual-tactile information in the primary sensory cortices S1 and V1 involved coordinated and directed firing of a small fraction of neurons, mainly PYRs, via direct cortico-cortical axonal projections.

## Discussion

Cross-modal modulation of neuronal assemblies in primary sensory cortices is necessary for multisensory processing. The present study provides insights into the cellular substrate of visual-tactile interactions by testing how individual neurons in S1 and V1 convey cross-modal stimuli in activity patterns along corticocortical axonal projections. We demonstrate that (1) in both S1 and V1, a small fraction of PYRs and INs respond to cross-modal stimuli; (2) visual-tactile stimulation augments the firing of INs and decreases the firing of PYRs (the most prominent effects were detected in S1 supragranular and granular layers and V1 granular and infragranular layers); (3) visual-tactile stimuli modulate the firing latency and sharpens the phase locking of both PYRs and INs to theta–beta band network oscillations; and (4) synchrony of spike trains coupling S1 and V1 via direct but sparse intercortical axonal projections increases after cross-modal stimulation.

New experimental findings of the last years profoundly challenged the traditional view on multisensory processing. Originally, it was assumed that the integration of inputs from different senses follows hierarchically organized pathways and mainly involves higher cortical areas and some subcortical nuclei (Meredith and Stein, 1983; Stein and Stanford, 2008; Reig and Silberberg, 2014). Accumulating experimental evidence, however, has documented cross-modal activation in primary sensory cortices, which traditionally have been considered as sensory specific (Ghazanfar and Schroeder, 2006). At the network level, sensory systems seem to share similar mechanisms of cross-modal integration. Modality-unspecific stimuli cause phase reset of ongoing spontaneous network oscillations, facilitating that modality-specific stimuli arrive during the same oscillatory phase (Lakatos et al., 2007; Sieben et al., 2013). As a result, the processing efficiency of stimuli augments (Fries et al., 2001). At the level of single neurons, most of the knowledge originates from studies in the auditory system. It has been reported that only a small number of individual neurons in the primary auditory cortex modify their spiking pattern in response to visual and tactile stimuli (Kayser et al., 2008; Kayser et al., 2010; Meredith and Allman, 2015). In the present study, we demonstrate that cross-modal modulation of neuronal firing takes place in S1 and V1 as well. Only a small fraction of both PYRs and INs changed their firing rate, and consequently, at population level, no significant differences could be detected. Detailed analysis of firing after uni- and bimodal stimulation enabled further categorization of these neurons and identification of subtle area-, layer-, and cell type-specific differences. In line with anatomic data of information processing along the sensory tract with direct thalamic inputs to both granular and infragranular layers (Meyer et al., 2010; Feldmeyer, 2012; Constantinople and Bruno, 2013), the largest proportion of unimodal PYRs and INs were detected in S1 and V1 granular and infragranular layers. In S1, the majority of PYRs and INs responded to both visual and tactile stimuli, yet their firing rate was mostly similar under all stimulation conditions. Only few neurons responded differently to bi- versus unimodal stimulation. The high prevalence of additive multisensory neurons confirms previous data showing that multisensory interactions in rodents are modulatory (Ghoshal et al., 2011). In V1, the distribution of PYRs and INs across different classes was similar to S1. In S1, cooccurrence of visual and tactile stimulation augmented interneuronal firing and decreased pyramidal firing in supragranular and granular layers. These effects are in line with the cross-modal suppression of neuronal firing described for visual-auditory and visual-tactile stimuli (Kayser et al., 2008; Sieben et al., 2013; Meredith and Allman, 2015). In V1, the decrease of pyramidal firing and increase of interneuronal firing were present in the infragranular layer, whereas an overall suppression of spiking was observed in the granular layer. Overall, these differences may indicate that subtle differences in the circuits entrained in cross-modal processing are specific for each primary sensory cortex, although the overall coding scheme is similar.

Complementary to changes in spiking rate, the temporal pattern of pyramidal and interneuronal firing was modulated. In S1, the spiking response latency for PYRs as well as in V1 for INs decreased in a similar way as previously reported for higher multisensory areas, such as superior colliculus (Rowland et al., 2007). By these means, the delay between sensory and motor activation equally decreases, improving behavioral performance (Frens et al., 1995; Goldring et al., 1996). Two scenarios might account for spike timing modulation in S1 before the onset of stimulus-induced firing in V1. First, visual-tactile information might either already be integrated at thalamic level from where it is fed forward to the neocortex, or S1 might receive inputs from matching or cross-modal first-order thalamic nuclei VPM and LGN (Cappe et al., 2009). It has recently been shown that visual stimulation excites VPM neurons, their firing onset preceding unimodal responses in S1 or V1 (Allen et al., 2016). In line with these findings, it was previously shown that network effects in S1 are visually modulated well before the onset of visually evoked responses in V1 (Sieben et al., 2013). Besides integrative processes at subcortical level, most likely thalamic, a second scenario implies the existence of cross-modal neurons in primary sensory areas that modulate spike timing in putatively unimodal sensory areas after bimodal stimulation. It has been demonstrated that sensory areas represent a heterogeneous pool of neurons responding not only to modality-specific stimuli but also to cross-modal inputs (Wallace et al., 2004; Meredith and Allman, 2015). The present data suggest that the same multifaceted neuronal responses occur in primary sensory cortices, which already have been shown to code complex information such as reward or locomotion (Niell and Stryker, 2010). Besides timing modulation of stimulus-induced firing, the number of phase-locked cells and the locking strength between pyramidal/interneuronal firing and theta–beta network oscillations increased across all layers in S1 and V1. Sharpening of spike timing seems to be an ubiquitary and efficient mechanism of multisensory processing (Bizley et al., 2007; Lakatos et al., 2007; Kayser et al., 2008). It correlates with the ability of cross-modal stimuli to reset the phase of theta–beta band network oscillations in primary somatosensory, visual, and auditory cortices (Lakatos et al., 2007; Sieben et al., 2013).

Single-cell responses to sensory stimuli are under the direct control of network states, such as anesthesia or sleep (Fontanini and Katz, 2008). Vice versa, the behavioral state affects circuit computations as well as long-range corticocortical interactions (Massimini et al., 2005; Ferrarelli et al., 2010; Fu et al., 2014; Kuchibhotla et al., 2017). The effects described here might have been modulated by urethane anesthesia that influences excitatory and inhibitory neuronal firing (Hara and Harris, 2002) and, consequently, might differ from multisensory interactions in the awake state (Iurilli et al., 2012; Ibrahim et al., 2016). Furthermore, it has been shown that urethane anesthesia strengthens thalamic burst firing which, in contrast to tonic firing mode, inhibits the transmission of sensory information to the cortex (Huh and Cho, 2013). By these means, the flow of unisensory and multisensory information to primary sensory cortex might be restricted under urethane anesthesia. At very high doses, urethane has been shown to evoke cross-modal responses in primary sensory cortices (Land et al., 2012) as well as increasing the prevalence of cross-modal neurons in modality-specific cortices (Lissek et al., 2016). While studies exploring multisensory processing in the anesthetized animal have found bimodal enhancement effects (Meredith and Stein, 1983; King and Palmer 1985), no bimodal enhancement but rather depressive effects of neuronal firing were found in the awake state (Populin and Yin, 2002). However, these disadvantages of sleep-like/anesthetized conditions should be related to the confounding effects of awake state (e.g., attention, diverse brain states) on multisensory processing at cortical level.

In the light of the presented findings, two major questions concerning the multisensory processing in primary sensory cortices need to be addressed. First, which circuits underlie the rate and temporal coding of multisensory information? Anatomic investigations revealed the existence of direct axonal projections between primary sensory cortices, although their density is very low (Sieben et al., 2013; Henschke et al., 2014). The analysis of layer-specific connectivity between S1 and V1 revealed that intercortically projecting neurons are mainly located in infragranular layers of S1 and V1 and are almost absent at the level of granular layer. This connectivity pattern differs from those reported for V1–A1 (Ibrahim et al., 2016). Corresponding to the distribution of projections, more V1–S1 spike trains in infragranular than in granular layer synchronized their firing. Most of intercortically connected neurons are PYRs and only very few are INs, confirming previous anatomic investigations on the distribution of long-range GABAergic connectivity (Tamamaki and Tomioka, 2010). Despite their low number, these neurons seem to have a high impact on multisensory processing. Hyperpolarization in supra- and infragranular layers of V1 were detected when A1 was activated by noise and most likely results from intercortical activation of infragranular neurons and a subsequent local infragranular-to-supragaranular inhibition (Iurilli et al., 2012; Ibrahim et al., 2016). Direct corticocortical connections might not be the only source of cross-modal inputs to modality-specific sensory areas. Primary sensory areas are located at the interface between subcortical thalamic relay stations and higher sensory areas. Feedback influences from multisensory convergence zones at the border of two sensory-specific cortices might send cross-modal inputs to primary sensory areas (Driver and Noesselt, 2008). In addition, direct connections have been identified between primary sensory cortices and association cortices in monkeys (Rockland and Ojima, 2003) and rats (Paperna and Malach, 1991; Sreenivasan et al., 2017). Particularly, the rat V1 has reciprocal connections with the temporal association cortex and extrastriatal areas (Miller and Vogt, 1984; Vaudano et al., 1991; Wang and Burkhalter, 2007; Laramée et al., 2011) that in turn send outputs to primary auditory areas (Smith et al., 2010). Whether similar connections exist between S1 and higher visual areas remains to be elucidated. However, it is questionable whether polysynaptic loops from one primary sensory area to another primary sensory area via higher sensory areas can account for fast multisensory effects described here. Besides the connectivity between primary sensory areas and higher sensory areas, sensory information might also be integrated already at the level of first-order thalamus from where it is fed forward to primary sensory cortices. Cross-modal neurons have recently been detected in VPM and audiovisual processing effects have been described in the medial geniculate body (Komura et al., 2005). Future investigations need to assess to which extend a similar wiring scheme and synaptic interactions account for the visual-tactile processing.

The second question with high relevance for understanding multisensory processing is to which extent rate versus temporal code complement each other for carrying cross-modal information. It has been suggested that changes in single neurons code for the discrete properties of sensory stimuli, whereas temporal code tags the relatedness of firing modulation to form a broader percept (Singer, 2009). While rate and temporal codes may act independently, the majority of studies proposed their dual action as key of information representation (Masuda and Aihara, 2007; Ainsworth et al., 2012; Panzeri et al., 2015). Synchronization of neural assemblies can be obtained using one or the other coding strategy. Coding by firing rate requires not only a large number of spikes and neurons but also homogeneous cell populations internally connected by equal weights. Therefore, its influence on assembly activity is rather limited and needs to be complemented by temporal coding that ensures that stimuli are timed to the optimal phase of network oscillations, thus having the increased salience. Our data revealed that both codes, rate and temporal, act simultaneously and underlie the communication between S1 and V1. Interestingly, the communication between S1 and V1 spike trains seems to be directed during cross-modal stimulation. Especially simultaneously recorded PYRs in the infragranular layer of S1 fire shortly before V1 neurons, suggesting that they drive the entrainment via monosynaptic projections.

In the light of present findings, we propose that cross-modal influences on early somatosensory or visual processing should improve the perception of tactile and visual stimuli. Neuronal firing precisely occurring during neuronal rhythms facilitates the transfer of information (Salinas and Sejnowski, 2001). While very few behavioral investigations addressed the S1–V1 interactions (Sieben et al., 2015), experimental evidence from auditory system supports this hypothesis (Alais and Cass, 2010; Gleiss and Kayser, 2012). Precise targeting and manipulation of neurons involved in interareal corticocortical communication is required in the future to understand the behavioral readout of their activity codes in multisensory perception.

## Synthesis

Reviewing Editor: Kate Jeffery, University College London Decisions are customarily a result of the Reviewing Editor and the peer reviewers coming together and discussing their recommendations until a consensus is reached. When revisions are invited, a fact-based synthesis statement explaining their decision and outlining what is needed to prepare a revision will be listed below. Note: If this manuscript was transferred from JNeurosci and a decision was made to accept the manuscript without peer review, a brief statement to this effect will instead be what is listed below.

The paper is well put together and almost ready for acceptance - there is a spelling mistake throughout (principle instead of principal for PCA). I wonder if the figures could be made less “busy” and more simple so that the take-home message is clearer - possibly by increasing the number. Font sizes could also usefully be increased.

Also, you refer throughout to “a small fraction” etc of neurons and it is not clear whether it is small compared to expected, or small but not zero - that is, is the point you are trying to make that there *are* such neurons or that there are not very *many* of them. It would help understanding to make this point early on (including the abstract).

Finally, the take-home message in the discussion and abstract could be made more general. What is the role of such cross-modal interaction; why should it be mainly inhibitory (to suppress competition and allow attentional focus perhaps?). Bear in mind that potential readers may not be familiar with such ideas about coupling and sensory integration.
